# OPCABG using right internal mammary artery and sequential saphenous vein graft in reverse sequence) in a PT with right pneumonectomy with compensatory hyperexpansion of the left lung

**DOI:** 10.1186/1749-8090-8-S1-O302

**Published:** 2013-09-11

**Authors:** A Zaheer, M Baghai, O Wendler

**Affiliations:** 1 Department of Cardiothoracic Surgery Kings College Hospital, London, UK

## Background

We report a case of OPCABG using RITA and sequential vein graft to PDA and OM2 (in reverse sequence) in a patient who had Right pneumonectomy and compensatory hyperexpansion of left lung resulting in mediastinal shift to the right.

## Case report

77 years old male with worsening angina and shortness of breath and diagnosed with 3 vessel disease and history of right pneumonectomy was referred for coronary artery bypass grafting.Other co morbidities included hypercholesterolemia, exsmoking, recurrent pneumonia, colorectal resection and chemotherapy for cancer and also had gastric ulcers and could not tolerate aspirin (hence not an ideal candidate for PCI).

CT chest was performed which showed heart to be deviated to the right side with tracheal deviation due to compensatory hyperexpansion of left lung.

Initial plan was to perform on pump CABG in view of single lung. However after midline sternotomy it was clear that the left lung dominated the chestand the heart lay posteriorly in the right hemi-thorax with slight rotation to theright. This made on-pump surgery difficult as there was no access to the right atrium and majority of the aorta lay behind the Pulmonary artery. Femoral venous cannulation would havebeen risky due to the right atrial shift at the level of the diaphragm. Decision was made to perform the procedure without cardiopulmonary bypass. The Right internal mammary artery was harvested in semi-skeletonised fashion and was anastomosed to the mid LAD. There was enough aorta accessible to place a single vein sequentially on the OM2 and PDA performing the top end to the aorta first and then OM2 anastomoses in side to side fashion followed by PDA anastomosesas end to side anastomosis. There were no periods of instability.

The postoperative course was uneventful apart from patient going into atrial fibrillation which was treated with amiodarone. Patient was discharged home on 6^th^ postoperative day.

